# Peripheral Nerve Surgery and Chemotherapy: A Review, Index, and Decision Tree for Nerve Surgeons Considering Neurotization After Oncological Nerve Injuries

**DOI:** 10.1227/neuprac.0000000000000206

**Published:** 2026-04-01

**Authors:** William ElNemer, Chase H. Foster, Muhammad Ali, Jawad Khalifeh, Ahmet Hoke, Daniel Lubelski, Allan J. Belzberg, Sami Tuffaha

**Affiliations:** 1 Department of Neurosurgery, School of Medicine, Johns Hopkins University, Baltimore, Maryland, USA;; 2 Department of Neurosurgery, George Washington University, Washington, District of Columbia, USA;; 3 Departments of Neurology and Neuroscience, Johns Hopkins University, Baltimore, Maryland, USA;; 4 Department of Plastic and Reconstructive Surgery, Johns Hopkins University, Baltimore, Maryland, USA

**Keywords:** Chemotherapy, Chemotherapy-associated neurotization failure, Chemotherapy-induced peripheral neuropathy, Nerve regeneration, Neurotoxicity, Peripheral nerve surgery, Oncology, Surgical decision-making

## Abstract

**BACKGROUND AND OBJECTIVES:**

Chemotherapy-induced peripheral neuropathy (CIPN) is a complication caused by some drugs used to treat cancers including metastatic spine and malignant nerve tumors. Debilitating denervation may result from such tumors directly or from their surgical resection. The inhibitory effects of adjuvant chemotherapy on nerve regeneration in peripheral neurotization surgeries are poorly understood and must be inferred from known mechanisms of CIPN. This narrative review aims to create an index of chemotherapeutics that may cause chemotherapy-associated neurotization failure (CANF) to better inform preoperative counseling, operative timing, and postoperative prognostication by peripheral nerve surgeons.

**METHODS:**

A narrative review of published English-language literature on CIPN was performed, focusing on its known pathogenic mechanisms and its clinical manifestations including onset, duration of dysfunction, and effect size. The literature was also searched for existing evidence on the effects of the same agents on nerve regeneration and outcomes after peripheral nerve surgery.

**RESULTS:**

CIPN can manifest with sensory, motor, and/or autonomic dysfunction. Platinum-based compounds, taxanes, vinca alkaloids, antimetabolites, and proteasome inhibitors are implicated drug classes, all of which have distinct neurotoxicity profiles. CIPN phenotypes vary in timing, severity, and nerve modalities affected. Some agents are associated with a “coasting” phenomenon, where neuropathy persists even after the offending drug's discontinuation. The neurotoxic milieu induced by chemotherapy likely impairs nerve regeneration, but this has not been addressed in the literature. Data on neurotoxic chemotherapy agents were synthesized and used to create an index and risk-stratified decision tree based on inferred likelihood of CANF.

**CONCLUSION:**

Denervated patients on neurotoxic adjuvant chemotherapy regimens pose unique challenges to peripheral nerve surgeons. At least a cursory understanding of CIPN-implicated drugs, mechanisms of neurotoxicity, presentations, and the inferred risk of CANF is recommended before pursuing neurotization surgery. This index serves as a resource for peripheral nerve surgeons in this clinical conundrum.

**ABBREVIATIONS:**
CANF: chemotherapy-associated neurotization failure
CIPN: chemotherapy-induced peripheral neuropathy.

Metastatic cancer frequently affects the spine and peripheral nervous system, often with significant morbidity. Approximately 10% of patients develop spinal lesions, and up to 25% experience neurological complications.^1,2^ Even after surgical resection of metastatic spinal tumors or malignant peripheral nerve sheath tumors (MPNSTs), persistent neurological deficits occur in 25%-30% of patients.^3,4^ Further neurological damage may arise from radical resections of aggressive lesions. These patients are often referred for peripheral nerve procedures such as neuroplasty, nerve grafting, or nerve transfers (“neurotization”).

To compound this problem, these patients are frequently prescribed chemotherapy, alone or adjuvant, targeted to the primary tumor biology. Chemotherapy-induced peripheral neuropathy (CIPN) refers to the dose-limiting neurotoxicity of chemotherapeutic agents,^5,6^ characterized by damage to the peripheral nervous system. It can present as sensory symptoms—including numbness, tingling, and pain in the extremities—but may involve motor symptoms, ataxia, or autonomic dysfunction.^6,7^ These impairments reduce quality of life and may necessitate treatment modification.^8,9^ The severity and duration of CIPN are influenced by the type of chemotherapeutic, cumulative dosage, and patient comorbidities.^5,6,10^ Although some patients recover after stopping chemotherapy, others experience prolonged or worsening symptoms, a phenomenon termed “coasting.”^11^

The effect of ongoing chemotherapy on nerve regeneration after neurotization remains unclear.^12,13^ Neurotoxic agents may impair nerve healing, leading to chemotherapy-associated neurotization failure (CANF). While delaying surgery until after chemotherapy or symptom resolution may mitigate CANF, doing so risks missing the critical window for functional recovery.^14,15^ Peripheral nerve surgeons must weigh these competing concerns. This narrative review provides an indexed and categorized overview of neurotoxic chemotherapies to aid operative timing, risk assessment, and patient counseling.

## THE SCOPE OF THE PROBLEM

CIPN refers to a dose-limiting neurotoxic complication of certain chemotherapeutic agents, characterized by peripheral nerve damage. It typically presents with sensory, motor, or autonomic dysfunction, most commonly with symmetric sensory loss, paresthesia, pain, and weakness in a “glove-and-stocking” distribution.^16,17^ Reported incidence rates varying from 30% to 80%, depending on the agent and dosage used.^5,6,18^ This excludes many otherwise ideal candidates who are receiving chemotherapy from peripheral nerve interventions.

Although sensory symptoms predominate,^17,19^ motor involvement may cause weakness, cramps, and impaired coordination.^7,20^ Less commonly, autonomic dysfunction leads to gastrointestinal dysmotility, orthostatic hypotension, and cardiac irregularities.^16,20^ These deficits are associated with poor quality of life and worse 5-year survival.^8,11,21^

### Chemotherapeutic Agents Implicated in CIPN

The pathophysiology of CIPN varies among different chemotherapeutics (Table). Platinum-based compounds, such as cisplatin and oxaliplatin, induce neurotoxicity through formation of DNA adducts, causing neuronal apoptosis.^20,33,34^ Taxanes (e.g., paclitaxel and docetaxel) disrupt microtubule dynamics which impair axonal transport and result in sensory neuropathy.^20,33,34^ Vinca alkaloids, such as vincristine, inhibit microtubule assembly causing axonal degeneration and neuropathic symptoms.^20,34^ Proteasome inhibitors, such as bortezomib, and immunomodulatory drugs, such as thalidomide, contribute to CIPN by impairing mitochondrial function and oxidative stress.^20,34^

**TABLE.** Index of Chemotherapeutics Groups by Drug Class That Contribute to Chemotherapy-Associated Neurotization Failure

Chemotherapeutic (generic)	Common US brand name(s)	Drug class	Mechanism(s) of toxicity	Typical dose (mg/dL)	Sensory neuropathy (incidence)	Motor neuropathy (incidence)	Autonomic neuropathy (incidence)	Coasting? (Y/N)	Duration of neurotoxic effects	Miscellaneous
*docetaxel*	Docivyx, Taxotere	Taxane (microtubule inhibitor)	Stabilizes microtubules by binding to β-tubulin, preventing depolymerization, inducing G2/M cell cycle arrest, and apoptosis	60-100 mg/m^2^ intravenously every 3 wk	11%-64%	4.4%-44%^15,22^	43.6%^15,22^	Y	9 wk, up to a decade in severe cases	1. Combination of docetaxel and carboplatin has lower neurotoxicity than the combination of paclitaxel and carboplatin 2. Higher potency and additional mechanism of actions such as lysosomal function compared with paclitaxel 3. Can be combined with gemcitabine as second line therapy for MPNSTs and other soft tissue sarcomas
*paclitaxel*	Abraxane, Taxol	Taxane (microtubule inhibitor)	Stabilizes microtubules by binding to β-tubulin, preventing depolymerization, inducing G2/M cell cycle arrest, and apoptosis	125-260 mg/m^2^ intravenously over varying cycle between weekly and once every 3 wk	47.2%-87%	17%-44%	13%-45.7%^15,23,24^	Y	3 mo, up to over a year in severe	1. Affects distal portions of sensory axons arrests axon growth without immediate axon fragmentation or cell death 2. Preclinical studies have shown associated nerve regeneration with paclitaxel
*vinblastine*	Velban	Vinca alkaloid (microtubule inhibitor)	Inhibits microtubule polymerization, causing mitotic arrest	6 mg/m^2^ intravenously weekly	5%-6%^25^	Very rare or unreported	Very rare or unreported	Y	3-6 mo, up to years in some cases	1. Metabolized by the cytochrome P450-CYP3A4 system 2. Reported loss of somatosensory sensation 3. Can cause difficulty with tasks requiring fine motor skills
*vincristine*	Citomid, Oncovin, Vincasar PFS, Vincrex	Vinca alkaloid (microtubule inhibitor)	Inhibits microtubule polymerization, causing mitotic arrest	14 mg/m^2^ intravenously weekly, maximum dose of 2 mg/m^2^ weekly	22%-78%^26,27^	23%-93%^26,27^	56%^26,27^	Y	2-12 mo, up to years in severe cases	1. Can cause cranial nerve palsies including facial nerve palsy and vocal cord dysfunction 2. Optic neuropathy has been reported 3. Seizures are rare but documented 4. Cognitive deficits and anxiety-like behavior has been observed in animal models 5. Bladder dysfunction has been reported especially in males
*vindesine*	Eldisine, Fildesin	Vinca alkaloid (microtubule inhibitor)	Inhibits microtubule polymerization, causing mitotic arrest	2-4 mg/m^2^ intravenously semiweekly to biweekly	30%-33%	30%-33%	No reported overall incidence but reported symptoms	Y	4 wk, up to several months	1. Other reported symptoms include myalgias, vertigo, diarrhea, skin pains, tinnitus, gastric pains, alopecia, and tremor 2. Reported cases of leukopenia and thrombocytopenia 3. Interacts with itraconazole to cause severe neurotoxicity and SIADH
*vinorelbine*	Navelbine	Vinca alkaloid (microtubule inhibitor)	Inhibits microtubule polymerization, causing mitotic arrest	25-30 mg/m^2^ intravenously weekly	24%-100%	24%-100%	20%^28,29^	Y	3-6 mo	1. Tetraplegia reported in a breast cancer patients 2. Combination vinorelbine and paclitaxel led to severe neurotoxic effects
*carboplatin*	Paraplatin	Platinum-based alkylating agent	Causes inter- and intra-strand DNA cross-links, disrupting replication and transcription	Dose (mg) = AUC × (GFR +25) Common target AUC: 4-6 mg/mL/min in most regimens	4%-10%	Very rare or unreported	Very rare or unreported	N	NA	Two genetic variants, rs56360211 near PDE6C and rs113807868 near TMEM150C, weakly associated with CIPN
*cisplatin*	Platinol	Platinum-based alkylating agent	Causes inter- and intra-strand DNA cross-links, disrupting replication and transcription	50-100 mg/m^2^ intravenously every 3-4 weeks 20 mg/m^2^ intravenously every 5 d	47%-71%	Very rare or unreported	Very rare or unreported	Y	1-4 mo, up to a few years	Can cause cognitive deficits, often referred to as “chemo-brain,” which includes problems with memory, attention, and executive function
*oxaliplatin*	Eloxatin	Platinum-based alkylating agent	Causes inter- and intra-strand DNA cross-links, disrupting replication and transcription	85 mg/m^2^ intravenously semiweekly	56%-92%	Very rare or unreported	Very rare or unreported	Y	2-3 months, up to 5 y	Acute neurotoxicity often manifests as cold-induced distal dysesthesia, paresthesia and muscular contractions that are worsened by exposure to cold
*cytarabine (cytosine arabinoside)*	Cytosar, Cytosar-U, Tarabine PFS	Antimetabolite (pyrimidine analog)	Mimics cytidine and incorporates into DNA, leading to chain termination and apoptosis	100 mg/m^2^/d by continuous intravenous infusion	1%-14%	Very rare or unreported	Very rare or unreported	N	NA	Can induce cerebellar neuronal damage, causing behavioral abnormalities and motor performance impairment
*methotrexate*	Trexall, Otrexup, and Jylamvo	Antimetabolite (folate analog inhibitor)	Inhibits dihydrofolate reductase, blocking the conversion of dihydrofolate to tetrahydrofolate	20 mg/m^2^ once weekly	Very rare or unreported	Very rare or unreported	Very rare or unreported	N	1-3 mo	Incidence of both central and peripheral neuropathy encompassing encephalopathy, leukoencephalopathy, or myelopathy more often than isolated peripheral neuropathy
*nelarabine*	Arranon, Atriance	Antimetabolite (purine analog)	Metabolized to an active purine analog and incorporates into DNA, leading to chain termination and apoptosis	Adults: 1500 mg/m^2^ intravenously 3 times in 1 week per month Pediatrics: 650 mg/m^2^/d intravenously for 5 consecutive days	11.5%-34%	4%-34%	Very rare or unreported	N	NA	Cases of GBS-like myelopathy
*bortezomib*	Velcade	Proteasome inhibitor	Inhibits the 26S proteasome, leading to the accumulation of misfolded proteins, which induces cellular stress and apoptosis	1.3 mg/m^2^ twice a week	34%-85%	2.6%-10%	8%^30-32^	Y	3-4 months, up to 8 mo	Incidence may be higher when used in conjunction with isoniazid, amiodarone, or HMG-CoA reductase inhibitors
*eribulin (eribulin mesylate)*	Halaven	Microtubule inhibitor	Inhibits microtubule polymerization, leading to mitotic arrest and apoptosis	1.4 mg/m^2^ intravenously on days 1 and 8 of a 21-d cycle	4%-32.4%	Very rare or unreported	Very rare or unreported	N	NA	1. Combination of gemcitabine and eribulin is less neurotoxic compared with the combination of paclitaxel and gemcitabine 2. It is also used as second line therapy for MPNSTs and other soft tissue sarcomas
*ixabepilone*	Ixempra	Semi-synthetic epothilone B	Binds to microtubules, promoting their stabilization and disrupting mitosis	40 mg/m^2^ intravenously every 3 wk	14%-67%	14%-23%	Very rare or unreported	N	NA	Combination with irinotecan can lead to delayed grade 3 peripheral neuropathy
*thalidomide*	Contergan, Neurosedyn, Talidex, Talizer, Thalomid	Immunomodulator	Combination of immunomodulation through the inhibition of TNF-α and modulation of cytokines, anti-angiogenesis through the inhibition of VEGF and FGF, direct effects on cancer cell survival and apoptosis, and interaction with cereblon which affects protein degradation pathways	100-300 mg orally daily, 400 mg/d for severe cases	25.2%-73%	16%-22%	Very rare or unreported	Y	1-3 mo	Can cause a reduction in sensory nerve action potential amplitude, a reliable marker for detecting early nerve damage

AUC, area under the curve; FGF, fibroblast growth factor; GBS, Guillain–Barré syndrome; GFR, glomerular filtration rate; HMG-CoA, 3-hydroxy-3-methylglutaryl-coenzyme A; MPNSTs, malignant peripheral nerve sheath tumors; NA, not applicable; PDE6C, phosphodiesterase 6C; PFS, preservative free solution; SIADH, syndrome of inappropriate antidiuretic hormone; TMEM150C, Transmembrane protein 150C; VEGF, vascular endothelial growth factor.

Various chemotherapeutic agents are associated with CIPN, each exhibiting unique neurotoxicity profiles. Although there are scant data on neurotoxic chemotherapeutics and their effects on nerve *regeneration*, a deleterious effect of some power can be reasonably inferred from the specific neuropathic effects highlighted in Table.

## CONTEXUALIZING THE PROBLEM

### Presenting to the Peripheral Nerve Surgeon

This review presumes an established oncological diagnosis with clinically confirmed neuron denervation; it is not intended to guide initial diagnostic workup. Patients undergoing active chemotherapy may present to peripheral nerve surgeons with lower motor neuron deficits resulting from tumors, resection, radiotherapy, or any combination thereof. These deficits may be compounded by CIPN, further impairing recovery. Surgical interventions often include neuroplasty, direct nerve repair, or, most commonly, distal nerve transfers.^35,36^ Resection of MPNSTs, complex sarcomas, and superior sulcus tumors often requires wide local excision,^3,37^ risking iatrogenic nerve injury of structures such as the brachial plexus. Similarly, en bloc spinal tumor resections may necessitate nerve root sacrifice, resulting in substantial motor and sensory deficits.^38^ Peripheral nerve reconstruction can restore function, with many patients achieving meaningful recovery.^39^ Some may also be referred for unrelated peripheral nerve injuries.

No human data exist on how chemotherapy affects nerve regeneration after neurotization. There is a single 1999 study that reported no significant difference in nerve regeneration after injury between control and chemotherapeutic (cisplatin and vincristine) groups in a rodent model, suggesting that nerve repair may be feasible despite concurrent or postoperative chemotherapy.^40^ In lieu of direct data, CIPN is used here as a surrogate for CANF.

Chemotherapeutic agents such as cisplatin, vincristine, and paclitaxel are known to cause significant neurotoxicity by causing axonal degeneration, myelin disruption, and reduced nerve conduction.^12,41^ Some agents such as eribulin and vinorelbine may permit better nerve recovery than the aforementioned agents.^12,41^ CIPN phenotypes vary in severity, duration, and affected modality. These same mechanisms likely impair regeneration after lower motor neuron injury, making CANF risk proportional to CIPN severity. Understanding the neurotoxic profiles of commonly used agents is critical for surgical planning and prognostication.

### Time Sensitivity

Nerve transfer surgery is most effective within 3 to 6 months of injury, as this window maximizes the odds of regenerating axons reaching denervated target muscle before irreversible atrophy and neuromuscular junction fibrosis.^14,42^ Delaying surgery significantly reduces the likelihood of functional recovery and must be weighed with the odds of success after peripheral nerve surgery. One study found that for every week of delay, the adjusted odds of achieving Medical Research Council strength grade ≥3 decreases by 3%.^43^

Although the exact timeline to Schwann cell quiescence and motor end plate degeneration varies, it is generally estimated at 12 to 18 months.^44^ In patients undergoing chemotherapy, this window may be even shorter. Thus, there is strong rationale for early intervention—even if neurotoxic chemotherapy is ongoing or CIPN symptoms persist.

Human studies using capsaicin-induced denervation show delayed and impaired regeneration,^45^ supporting the notion that preexisting neuropathy or other predispositions further hinder recovery. For these reasons, surgery within the 6-month window remains prudent, regardless of patient demographics or pharmacological context, to maximize the likelihood of successful reinnervation (Figure). An interactive, web page version of Figure can also be accessed at this URL: https://wgelnemer.github.io/CANF/CANF%20Tree.html.

**FIGURE.**
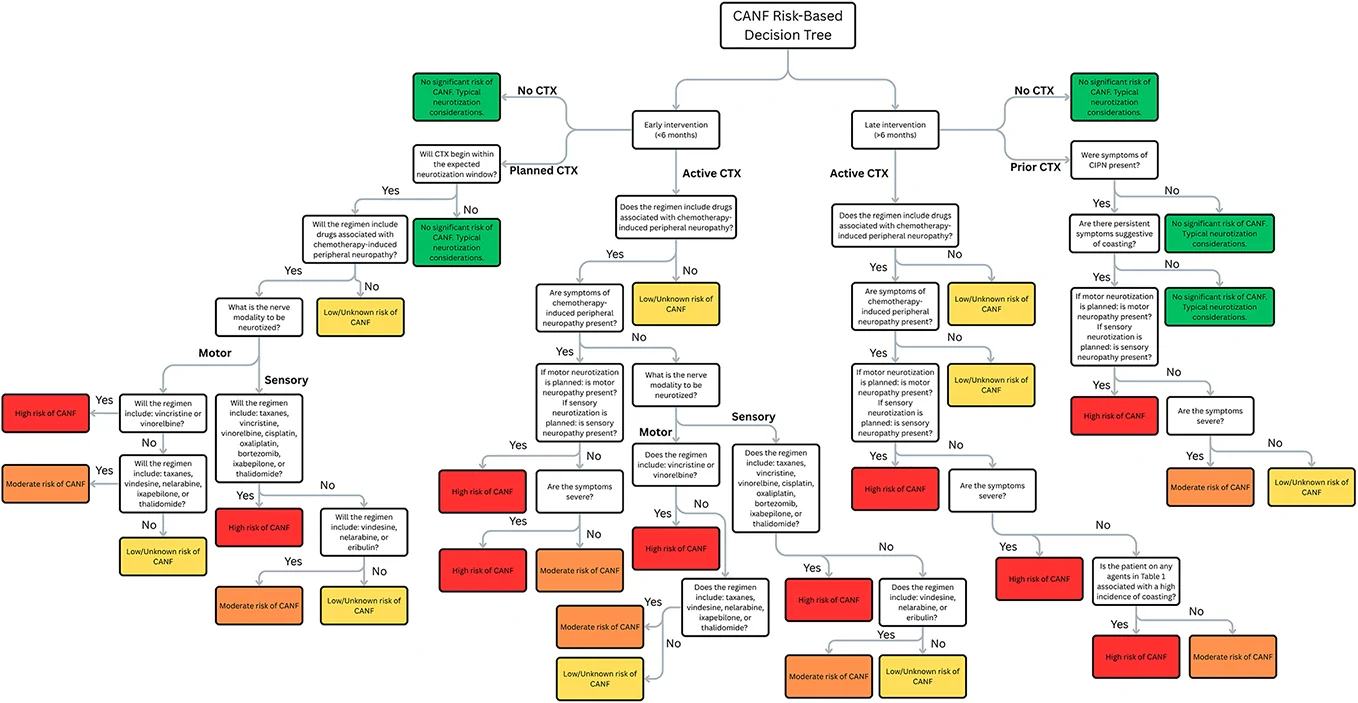
CANF Risk-Based Decision Tree showing an algorithm for aid in clinical decision-making in patients at risk for CIPN due to chemotherapeutic treatment. CANF, chemotherapy-associated neurotization failure; CIPN, Chemotherapy-induced peripheral neuropathy; CTX, chemotherapy.

### Neurotoxicity Phenotypes

Details on the duration and coasting potential of various chemotherapeutics are summarized in Table. CIPN manifests in 3 distinct patterns:

1. Rapid Onset: Some patients experience a sudden and severe onset of CIPN shortly after starting treatment. Symptoms may worsen if the medication is continued but may improve if it is discontinued. This pattern is commonly associated with drugs such as paclitaxel and oxaliplatin, with symptoms often peaking within days of administration.^15,46^
2. Gradual Onset: In other cases, CIPN develops progressively over the course of treatment. This gradual or insidious onset is marked by increasing numbness, tingling, and pain, typically occurring in a stocking-glove distribution. It is frequently linked to many neurotoxic chemotherapy agents.^11,15,47^
3. Delayed Onset: Some patients do not exhibit CIPN during treatment but develop symptoms weeks or months after the chemotherapy has ended during the “coasting” phenomenon, when neuropathy progresses despite cessation of the offending chemotherapeutic drug. This delayed onset is particularly noted with oxaliplatin, where neuropathy may worsen for 2-3 months post-treatment before showing signs of improvement.^15,48^

## ADDRESSING THE PROBLEM

Absence of CIPN symptoms does not guarantee low neurotoxicity risk, given variability in presentation. Subclinical or delayed-onset neuropathy (“coasting”) may still occur. Surgical planning should consider the specific CIPN phenotype: rapid onset or severe cases may warrant caution, while gradual or delayed onset may permit earlier intervention.^49,50^ Reviewing the chemotherapeutic regimen is essential, as taxanes and platinum compounds carry high CIPN risk.^47,50,51^ Neurotization urgency should be balanced against CIPN risks, with close monitoring and possible chemotherapy dose adjustments if surgery cannot be delayed.^11,15^ American Society of Clinical Oncology guidelines recommend modifying chemotherapy dosing for CIPN patients, emphasizing a patient-centered approach to balance treatment benefits, CIPN risks, and surgical needs.^15^

For low-risk patients—based on phenotype and regimen—nerve surgery during chemotherapy may be reasonable if the agent has low or no known motor toxicity.^48,52^ Conversely, high-risk patients or those referred early may benefit from delaying surgery until chemotherapy and the “coasting” window have passed.^12,48^ This decision must be weighed against the risk of missed reinnervation opportunities.

Agents with lower motor neuropathy risk may be less likely to impair motor neurotization, and the same may apply to sensory nerves. Some agents (e.g., vinblastine, cytarabine, eribulin) predominantly affect a single modality, while others cause multimodal neuropathy (Table). Whether this modality specificity extends to regeneration remains unknown.

In selected cases, surgeons may cautiously proceed with neurotization if the chemotherapy cocktail is associated with a low incidence of the relevant neuropathy, although granular data are lacking. There are no granular published data to guide these assumptions. To aid risk stratification, we developed a decision tree (Figure) informed by the index that may help nerve surgeons stratify the risk of CANF.

The underlying algorithm makes several assumptions and arbitrary categorizations based on real data reported in Table. CANF risk is categorized as high, moderate, or low/unknown. If the upper range of modality-specific CIPN incidence exceeds 50%, risk is considered high. If between 11% and 50%, it is moderate; if ≤10%, it is low/unknown.

Five additional points of logic underlying the decision tree branches are as follows.

1. Even if no CIPN symptoms are present for patients on active chemotherapy, CIPN may still develop during the neurotization window.
2. Lack of known neurotoxic agents does not eliminate CANF risk, so these are conservatively categorized as low/unknown.
3. Severity assessment should be performed ad hoc by the surgeon and patient, and it is both partly objective and subjective.
4. Four, coasting is only considered along the active chemotherapy pathway for late (i.e., >6 months) interventions because the residual or ongoing deficits may suggest a prolonged period of inhibition of neural regeneration, compounding the already unfavorable prognosis for reinnervation success secondary to the delayed presentation.
5. Only decision branches with no ongoing effect from chemotherapy recommend “typical neurotization considerations”-not because outcomes are improved but because CANF risk is neutral.

There is certainly a possibility that chemotherapy could have been previously completed in the “early intervention” branches and that there may be planned chemotherapy in the “late intervention” branches. For simplicity, these possible progressions were not included in the figure; however, they are readily interchangeable.

## CHEMOTHERAPEUTIC AGENTS AND MECHANISMS OF NEUROTOXICITY

This section outlines therapies a peripheral nerve surgeon may encounter when treating CIPN. All neurotoxicity profiles derive from “level A” data from classes 1 and 2 studies, except for methotrexate, whose peripheral effects are rare.

### Taxanes

Taxanes are among the most neurotoxic chemotherapeutics. Paclitaxel and docetaxel stabilize microtubules, preventing depolymerization, disrupting mitosis, and inducing apoptosis. This impairs axonal transport and leads to neuropathy.^51,53^ Therefore, their use is usually limited to metastatic presentations of taxane-sensitive primaries (e.g., breast, ovarian, lung, and prostate).^54^

#### Docetaxel

Docetaxel mirrors paclitaxel's toxicity but with lower CIPN rates, ranging from 11% to 64%.^55,56^ Sensory and motor deficits may persist for up to 2 years, with coasting reported.^57^ Treatment approaches mirror those for paclitaxel.^15,22^ Of note, the combination of the pocetaxel/carboplatin resulted in similar survival measures and quality of life scores as paclitaxel/carboplatin, but with lower rates of neurotoxicity.^58^

#### Paclitaxel

Paclitaxel is strongly associated with sensory CIPN in 47%-87% and motor symptoms in ≤15%.^15,23,24^ Symptoms are often reversible, although coasting is common; recovery may take up to 12 months.^12,15,17,59^ Management strategies for CIPN include dose reduction, cessation, and pharmacological interventions such as duloxetine, which has demonstrated efficacy in reducing symptom severity.^15,22^ Despite treatment, chronic CIPN can persist, with up to 30% reporting long-term neuropathic pain.^60^ While neuropathic pain of this etiology may not be addressed by peripheral nerve surgery, restoration of critical sensory fields, such as the palmar and plantar surfaces, may be particularly inhibited by taxanes.

### Vinca Alkaloids

This class includes vincristine, vinblastine, vindesine, and vinorelbine. These agents destabilize microtubules, prevent tubulin polymerization, thus disrupting mitotic spindles and axoplasmic transport and thus leading to neurotoxicity.^26^ Use is largely limited to hematological and lymphatic cancers and occasionally pediatric glial neoplasms.^61^

#### Vinblastine

Vinblastine is less neurotoxic than vincristine, with reported CIPN incidences of 5%-6%.^25^ Sensory symptoms are common, while motor and autonomic effects are rare.^25^ Recovery usually occurs quickly within 6 months.^25,48^

#### Vincristine

Vincristine is highly neurotoxic; most patients experience CIPN.^26^ Features include motor neuropathy, sensory neuropathy, and unique autonomic effects such as constipation and orthostatic hypotension.^27^ Severe neuropathy is dose-dependent, with cumulative doses above 2 mg/m^2^ possibly causing irreversible damage.^15^ Chronic symptoms often persist despite dose limitation.^15,62^

#### Vindesine

CIPN incidence ranges from 30% to 33%, mainly encompassing sensory modalities.^28^ Unlike vincristine, vindesine symptoms are milder and more likely to resolve within a few months after treatment cessation.^28^ Dose reduction is common to mitigate neurotoxicity.^15^

#### Vinorelbine

Vinorelbine has been shown to be less toxic than vincristine and vindesine, yet a 1996 study reported 100% of peripheral neuropathy.^28,29^ The onset of neurotoxicity is often delayed, with symptoms peaking several months after treatment and resolving after 3-6 months.^63^

### Platinum-Based Alkylators

Oxaliplatin, cisplatin, and carboplatin form intra-DNA and inter-DNA cross-links, impairing replication and inducing neuronal apoptosis.^64^ These agents are most commonly used in germ cell tumors and certain central nervous system neoplasms such as medulloblastomas but are occasionally employed in the treatment of refractory or metastatic MPNSTs.^65,66^

#### Carboplatin

Carboplatin has a lower incidence of neurotoxicity compared with cisplatin, with sensory neuropathy in 4%-10% of cases.^58,67^ Although associations are weak, genetic variants, such as single-nucleotide polymorphisms near Phosphodiesterase 6C and Transmembrane protein 150C, may predispose patients to carboplatin-induced CIPN.^68^

#### Cisplatin

Cisplatin causes sensory neuropathy in 47%-71% of patients. Motor and autonomic neuropathies are rare.^69^ Coasting is common, with symptoms persisting for 1-4 months and occasionally longer.^69,70^ Chronic neuropathy and cognitive deficits may also develop.^69,70^

#### Oxaliplatin

Oxaliplatin causes sensory neuropathy in 56%-92% of patients.^71^ Acute, cold-triggered symptoms resolve in days, while chronic symptoms may persist for years.^72^ Coasting, notably, is rare unlike the other platinum-based compounds.^72^ Treatment includes duloxetine and calcium/magnesium infusions.^72^ Its distinctive cold sensitivity likely stems from sodium channel effects.^73^

### Antimetabolites

Antimetabolites such as cytarabine, nelarabine, and methotrexate mimic nucleotide analogs, disrupting DNA synthesis leading to apoptosis.^74^ They are mainstays of treatment for solid tumor metastases and hematological malignancies, often in conjunction with platinum-based agents to increase efficacy.^75^ The nerve surgeon may encounter methotrexate in particular even outside of a cancer context due to its use in rheumatological and auto-immune disorders.

#### Cytarabine

Cytarabine is a pyrimidine analog used in hematological malignancies. Sensory neuropathy is reported in 1%–14% of patients.^76^ Coasting is not observed with cytarabine. High doses may induce cerebellar toxicity, leading to behavioral abnormalities and impaired motor performance.^76,77^

#### Methotrexate

Methotrexate is a folate analog that inhibits dihydrofolate reductase, blocking DNA synthesis. Peripheral neurotoxicity is rare and typically seen with high-dose oncological use. Central neurotoxicity—encephalopathy or myelopathy—is more common.^78^ Data on peripheral effects are limited to sparse case reports of cranial or spinal polyradiculopathy, likely placing the actual incidence of peripheral neuropathy to <1%.^79^

#### Nelarabine

Nelarabine is a purine analog that is associated with sensory neuropathy in 12%-34% and with motor neuropathy reported in 4%-34% of patients.^80^ Rarely, Guillain–Barré syndrome–like myelopathy have been reported.^80,81^

### Miscellaneous Chemotherapeutic Agents

The following chemotherapeutics do not fall in the previous classes and will be described individually.

#### Bortezomib

Bortezomib, a proteasome inhibitor, disrupts protein homeostasis by inhibiting the 26S proteasome, leading to cellular stress and apoptosis.^30^ Sensory neuropathy occurs in 34%-85% of patients, while motor neuropathy occurs in 3%-10% of patients.^31^ Coasting may persist for up to 8 months in severe cases.^32^ Its neurotoxicity may increase when used alongside isoniazid or amiodarone.^32^

#### Eribulin

Eribulin, a microtubule inhibitor, prevents microtubule polymerization, causing mitotic arrest and apoptosis.^82^ Sensory neuropathy is reported in 4%-32% of patients; motor and autonomic neuropathies are rare.^83^ Combinations with gemcitabine reduces neurotoxicity compared with paclitaxel.^84^

#### Ixabepilone

Ixabepilone is a semisynthetic epothilone B that stabilizes microtubules and disrupts mitosis.^85^ Sensory neuropathy is reported in 14%-67% of patients; motor neuropathy occurs in 14%-23%.^83,86^ Combining ixabepilone with irinotecan may lead to delayed grade 3 peripheral neuropathy.^87^

#### Thalidomide

Thalidomide is an immunomodulator that inhibits TNF-α, modulates cytokines, and exhibits antiangiogenic effects.^88^ Sensory neuropathies occur in 25%-73% of patients; motor neuropathies occurs in 16%-22% of patients.^89^ Coasting can last for 1-3 months.^90^ It may reduce sensory nerve action potential amplitude, indicating early nerve damage.^91^

### Immunosuppression

Some immunosuppressive agents, such as tacrolimus, may enhance nerve regeneration via immunophilin-dependent pathways distinct from its immunosuppressive effects.^92^ In vivo studies demonstrate that systemic administration accelerates axonal regeneration and functional recovery by up to 29% with optimal dosage at 5 mg/kg in rats.^93^ Other agents with neuroregenerative potential include cyclosporine, rapamycin, nonimmunosuppressive immunophilin ligands (e.g., FK1706), dexamethasone, and artesunate, each demonstrating variable success in experimental models.^94^ However, their clinical translation remains highly limited by safety concerns and lack of large-scale trials in humans. Chemotherapy-induced immunosuppression itself (due to cytotoxicity of hematopoietic progenitor cells and CD4^+^ T-cell depletion)^95^ is unlikely to cause a paradoxical increase in nerve regeneration, but this has not been empirically disproven.^96^

## CONCLUSION

Metastatic spinal disease or nerve tumors can directly or indirectly (i.e., surgically) cause neurological deficits that can be improved by neurotization. However, concurrent neurotoxic chemotherapy complicates decisions about surgical timing.^12,41^ Systemic and neurotoxicity can create significant challenges for patients with cancer, profoundly affecting their quality of life, delaying or preventing reinnervation if surgery is pursued, and increasing complications such as wound healing.^49,97^

While deferring surgery until chemotherapy and coasting resolve is ideal, delays may miss the window for functional recovery. Surgeons must weigh the benefits of timely neurotization against ongoing systemic toxicity.^15,48^ Therefore, this narrative review and accompanying algorithm (Figure) and index (Table) can be referenced when faced with this conundrum and may help to both guide preoperative decisions and provide more informed patient counseling.

Further research is needed to explore whether countermeasures for neurotoxicity associated with chemotherapy, such as pharmacological interventions^98^ or neuromuscular training,^99^ could be used to supplement peripheral nerve surgery and mitigate the adverse effects of neurotoxicity on nerve regeneration. In the absence of direct data, a pragmatic approach that integrates epidemiological, pharmacological, and surgical knowledge with clinical experience offers the best framework for individualized patient care. A tailored treatment strategy—grounded in an understanding of CIPN phenotypes, the neurotoxic profiles of chemotherapies, and the parameters of peripheral nerve surgery—is essential for optimizing outcomes in this uniquely challenging population.
